# In situ–Directed Growth of Organic Nanofibers and Nanoflakes: Electrical and Morphological Properties

**DOI:** 10.1007/s11671-010-9758-z

**Published:** 2010-08-25

**Authors:** Roana Melina de Oliveira Hansen, Morten Madsen, Jakob Kjelstrup-Hansen, Horst-Günter Rubahn

**Affiliations:** 1 NanoSYD, Mads Clausen Institute, University of Southern Denmark, Alsion 2, 6400, Sønderborg, Denmark

**Keywords:** Organic semiconductors, In situ growth, Organic nanofibers

## Abstract

Organic nanostructures made from organic molecules such as *para*-hexaphenylene (*p*-6P) could form nanoscale components in future electronic and optoelectronic devices. However, the integration of such fragile nanostructures with the necessary interface circuitry such as metal electrodes for electrical connection continues to be a significant hindrance toward their large-scale implementation. Here, we demonstrate in situ–directed growth of such organic nanostructures between pre-fabricated contacts, which are source–drain gold electrodes on a transistor platform (bottom-gate) on silicon dioxide patterned by a combination of optical lithography and electron beam lithography. The dimensions of the gold electrodes strongly influence the morphology of the resulting structures leading to notably different electrical properties. The ability to control such nanofiber or nanoflake growth opens the possibility for large-scale optoelectronic device fabrication.

## Introduction

Optoelectronic devices are abundant in our electronics technology including for example LEDs, lasers, and photodetectors. For miniaturized, on-chip applications such as optical detection in lab-on-a-chip systems, integration of optoelectronic functionality [1] with silicon-based technology is of high interest due to the advanced technologies available for silicon processing. Optoelectronic functionality, however, is often difficult to achieve using only silicon technology due to silicon's indirect band gap. One possible solution is to integrate unconventional materials into platforms fabricated by means of conventional silicon technology, such as materials being organic thin films or nanofibres or inorganic nanowires with direct band gaps [2-5].

Much research has focused on inorganic nanowires, both in terms of proof-of-principle optoelectronic devices like LEDs [6], lasers [7], and photodetectors [8] and also in terms of large-scale integration methods such as transfer printing [9] and in situ growth [10]. The organic counterparts, however, have so far received less attention despite several unique advantages such as inherent tunability by the ability to tailor molecules for specific applications through synthetic chemistry [11,12], relatively low material cost, low temperature processing, and potentially high efficiency [13]. In addition to these two groups of nanowires, also hybrid (metal/organic or metal-oxide/organic) nanowires have been the subject of much study due to their potential within for example nanoelectronics [14] and energy storage [15] applications.

In this work, we focus on organic nanostructures. Initial growth experiments have demonstrated that *para*-hexaphenylene (*p*-6P) molecules can self-assemble into mutually parallel aligned nanofibers when deposited by physical vapor deposition on a globally [16] or locally [17] heated muscovite mica substrate. The resulting fibers are straight, crystalline and have specific optical properties such as emission of polarized blue light [18], waveguiding [19], and lasing [20]. However, muscovite mica is not further processable, making it difficult to pattern device architecture directly on the growth substrate. One solution to this problem is to transfer the nanofibers to an appropriate device platform. However, this method is complicated due to the weak van der Waals forces between the molecular constituents that render the nanofibers fragile, and thereby make it difficult to directly adopt the printing transfer techniques developed for large-scale transfer of inorganic nanowires [9].

An alternative surface for *p*-6P nanofiber growth is that of a thin gold film. However, the resulting fibers do not present the mutual alignment observed for nanofibers grown on mica [21]. An option is to use gold-coated silicon as substrate, which can be processed to have micro- or nanoscale surface structures. We have recently shown that nanofibers grown on microstructured, gold-coated substrates tend to grow perpendicular to the structures and that orientation and length control can be achieved [22]. In a further development of this method, we showed that it is also possible to grow fibers between nanoscale metal structures that can guide the nanofiber growth [23].

In this work, we demonstrate in situ growth of nanostructures (nanofibers and nanoflakes) between electrodes on a device platform to directly establish the necessary electrical connection that enables the use of organic nanostructures as optoelectronic device components. We have fabricated transistor platforms by preparing gold drain and source electrodes on silicon dioxide substrates, using the underlying silicon as backgate. With this approach, the nanostructures can be grown directly on the gold electrodes, bridge small gaps, and establish electrical contact. We show how the *p*-6P nanostructure morphology depends on the electrode design for this in situ growth, and how this affects the electrical characteristics.

## Experimental

The device platform is patterned in a mix-and-match process, where bonding pads and connecting electrodes are first fabricated on 100 nm silicon dioxide on highly doped silicon by optical lithography, metal deposition (5 nm Ti/30 nm Au), and lift-off. Nanoscale drain and source gold electrodes, partly overlapping the previously fabricated connecting electrodes, are then fabricated by e-beam lithography (EBL), gold deposition and lift-off. The electrode pattern is written by EBL at an acceleration voltage of 30 keV in 150 nm of PMMA resist. After exposure, the pattern is developed by immersion in a 3:1 IPA:MIBK solution, followed by metal deposition of (2 nm Ti/30 nm Au) and lift-off. Finally, the substrates are cleaned in a mild oxygen plasma to remove any organic residues, and the substrates are then ready for nanofiber growth.

Organic elongated *p*-6P nanostructures are grown on the nanostructured gold electrodes by physical vapor deposition of *p*-6P molecules at a rate of 0.1 Å/s from a Knudsen cell. The nominal *p*-6P thickness is 20 nm as measured by a cooled quartz microbalance. During deposition, the substrate is heated to 185°C, and the deposition is performed under high vacuum conditions (10^-9^ mbar).

The electrical characterization is made using a custom-built, LabVIEW-controlled measurement set-up, where the voltage is controlled by a 16-bit National Instruments DAQ card, which also samples the current measured by a Stanford Research SR 570 current amplifier. The dimensions of electrodes and nanofibers are characterized by scanning electron microscopy (SEM) and atomic force microscopy (AFM) operated in tapping mode to avoid damage to the nanofibers.

## Results and Discussion

**Figure 1:**
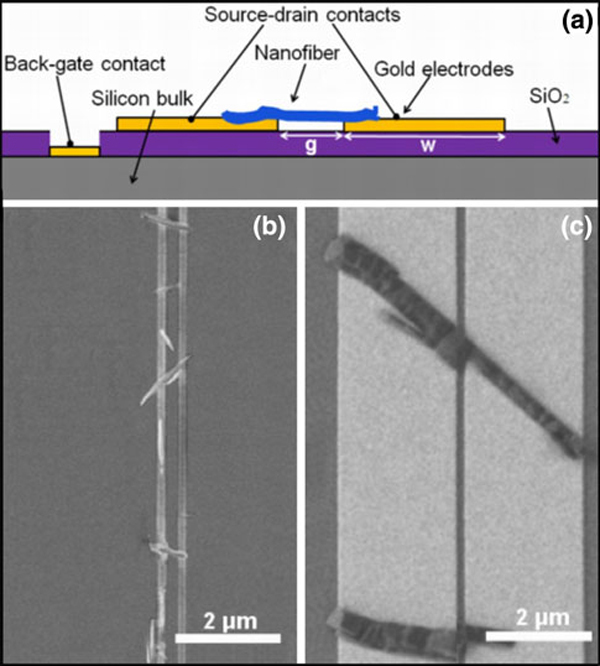
**a Schematic drawing of the device platform**. **b**, **c** SEM images of gold electrodes with *g* = 200 nm and *w* = 150 nm (**b**) and 2.4 μm (**c**) after *p*-6P deposition

**Figure 2:**
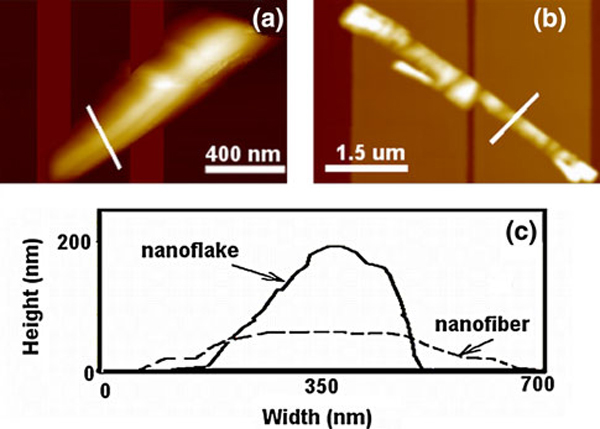
**a, b AFM images of gold electrodes with *g* = 200 nm and a *w* = 150 nm (nanoflakes) and b 2.4 μm (nanofibers) after *p*-6P deposition**. The cross-sectional profiles of both organic structures are shown in (**c**)

In order to investigate how the electrode design influences the resulting nanostructures, pairs of drain and source gold electrodes were fabricated with the separation gap *g* varying from 150 nm to 1 μm and the electrode width *w* between 150 nm and 2.4 μm; see Figure 1a. Upon deposition of *p*-6P molecules, elongated organic nanostructures are formed on the gold surfaces and in most cases bridge the gaps, thereby establishing electrical connection. Figure 1b,c shows SEM images of nanostructures grown on electrodes with two different widths at a gap of 200 nm. The AFM images and cross-sectional profile of each of the *p*-6P structures are shown in Figure 2. In both cases, the *p*-6P nanostructures are bridging the gaps; however, there is a difference in the morphology of structures depending on the electrode width. Narrow gold electrodes (Figure 2a) lead to tall and narrow nanostructures (here termed "nanoflakes"). This type of structures is also observed for nanostructures formed on alumina templates without gold coating [24]. Wider electrodes (Figure 2b) lead to fiber-like nanostructures ("nanofibers") more resembling those grown on plane (non-structured) gold surfaces.

The molecular orientation in the grown nanostructures was checked by polarization-dependent optical measurements. The luminescence output from a *p*-6P molecule is aligned along the long molecular axis [16]. Fluorescence microscopy showed that both kind of structures (nanofibers and nanoflakes) emit light polarized approximately perpendicular to the long axis of the nanostructures, which demonstrates that the molecules are oriented in this direction. This indicates that the molecular orientation of both nanofibers and flakes is similar to that known for *p*-6P nanofibres grown on muscovite mica.

**Figure 3:**
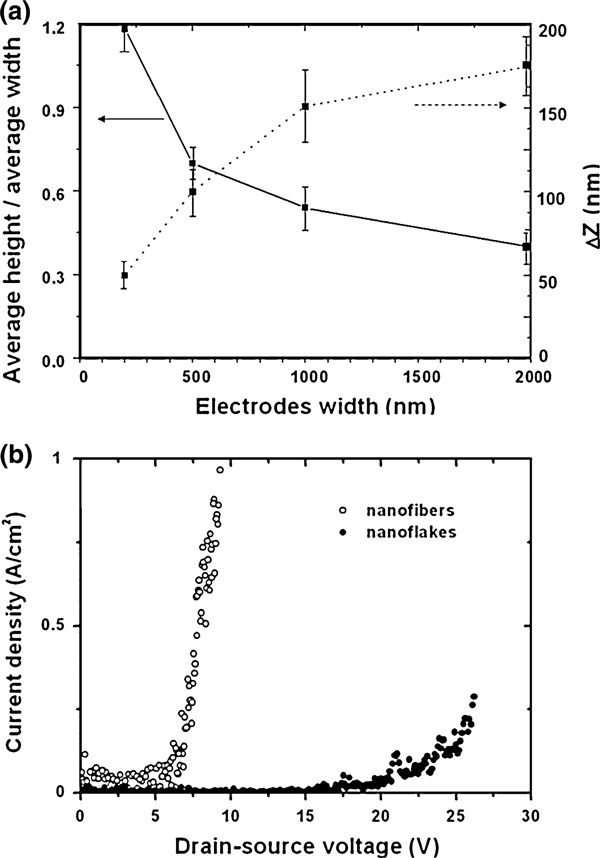
**a Ratio between average height and average width for the structures grown on electrodes with different widths are plotted as a *solid line* in the graph**. Δ*Z* factor for the structures grown on electrodes with different widths are plotted as a *dashed line* in the graph. **b** Electrical characteristics for *p*-6P nanostructures grown on gold electrodes with *w* = 200 nm (nanoflakes) and *w* = 2 μm (nanofibers)

A systematic investigation of the morphology of the organic nanostructures was made for different electrode widths *w*, by measuring the dimensions (width and height) of the grown nanostructures by SEM and AFM, respectively, and by calculating the aspect ratio, i.e. height-to-width ratio, as well as the difference between the maximum and minimum heights (Δ*Z* = *Z*_max_ - *Z*_min_) along the long axis of each individual structure. Figure 3a shows how these factors change as a function of the electrode width *w*. The data are based on measurements on about 120 nanostructures. For wide electrodes, the ratio between the average height and the average width of the nanostructures is smaller than for structures grown on narrow electrodes. We propose the following explanation for the phenomenon: on wide electrodes, the surface diffusion area is large, and *p*-6P clusters can aggregate and form long and flat fibers. On narrow electrodes, the surface diffusion area, where clusters are formed, is smaller, and the growth is hindered by boundaries. As a consequence, the molecules grow preferentially side by side in the vertical direction since tail-to-tail growth for rod-like molecules is improbable. The structures grown on wide electrodes exhibit a more jagged morphology, as previously shown for *p*-6P nanofibers grown on gold surfaces [21,22]. This effect is also shown in Figure 3a, which shows an increase in Δ*Z* for structures grown on wider electrodes.

Figure 3b shows electrical measurements made by applying a voltage between the drain–source electrodes and measuring the current flowing through the organic nanofibers/flakes grown on narrow (*w* = 200 nm) and wide (*w* = 2 μm) electrodes, respectively, with zero volts applied to the gate. Measurements prior to *p*-6P deposition were performed to verify that the measured current is not related to any leakage current. The current density values were calculated by dividing the measured current with the total nanofiber/nanoflake cross-sectional area. The curves confirm that it is possible to establish direct electrical connection to organic nanostructures and shown here is a clear difference in the electrical characteristics for the different structures with significantly higher current densities in the nanofibers even at relatively low voltages.

**Figure 4:**
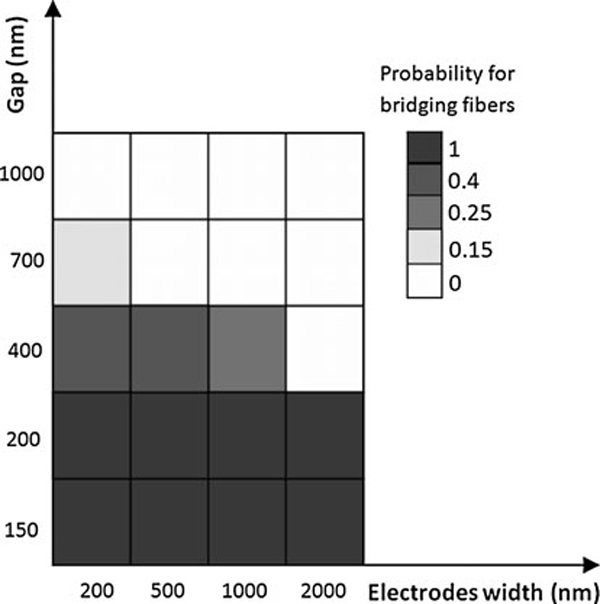
**Probability for having at least one bridging *p*-6P structure on a device with varied electrode widths and separation gaps**.

In order for a field-effect transistor to reach saturation, short-channel effects should be avoided by having a channel length that is at least 10 times larger than the gate oxide thickness [25,26]. All experiments presented here have been made on substrates with 100 nm oxide, so in order to minimize short-channel effects, the channel length i.e. the gap to be bridged should be increased to 1 μm. The first experiments on platforms with gaps of these dimensions showed that the nanoflakes or nanofibers are not able to bridge such long gaps. In order to determine the maximum gap that the organic structures can bridge, a set of devices with varied electrodes widths *w* and separation gaps *g* were fabricated and investigated. Figure 4 illustrates the probabilities for the organic nanostructures to bridge at different configurations. The data were extracted from around 150 devices, and it shows the probability for the device to have at least one bridging structure. For *g* < 200 nm, the probability for bridging structures is 1, but it decreases when *g* increases, and it is 0 for *g* = 1 μm. Therefore, the chosen standard separation gap was 200 nm.

It should be noted that growth parameters such as the substrate temperature during deposition and the nominal *p*-6P thickness have a large influence on the probability for the nanostructures to bridge the gaps. In the present experiments, the substrate temperature was kept constant at 185°C and the nominal thickness was 20 nm. It was observed that there was a high probability for the nanostructures to bridge the gaps under these conditions. At even higher surface temperatures, no nanostructure growth was observed.

**Figure 5:**
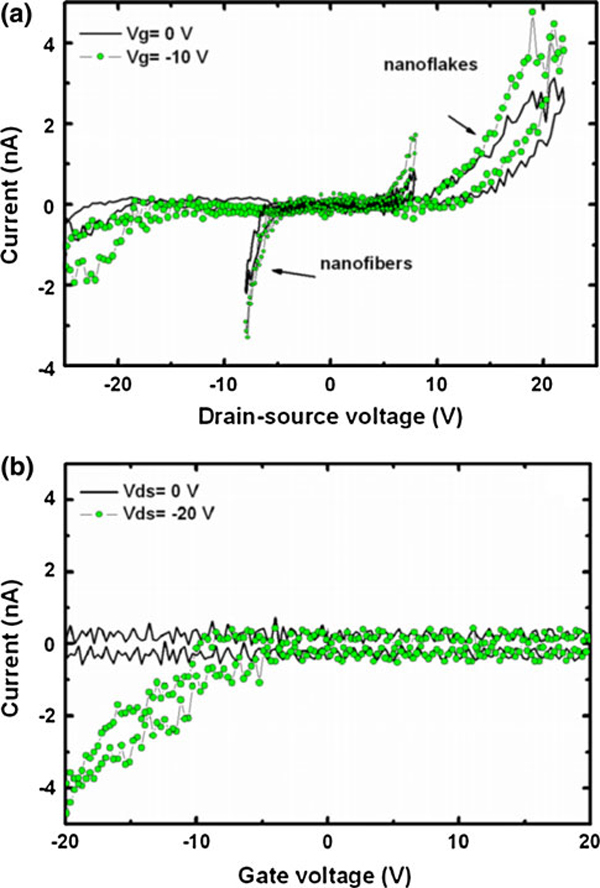
**a Output curves for devices containing nanoflakes (*w* = 200 nm) and nanofibers (*w* = 2 μm)**. **b** Transfer curves for the nanoflakes device. The oxide thickness is 100 nm and the drain–source separation gap is 200 nm

More elaborate results of the electrical characterization are shown in Figure 5a, which include output characteristics (*I*_DS_ vs. *V*_DS_) for narrow (*w* = 200 nm) and wide (*w* = 2 μm) electrodes. These were measured by sweeping the drain–source voltage from 0 V to positive voltages, back to negative voltages, and returning and finishing the measurement at 0 V. Each sweep was performed with a constant gate voltage (0 or -10 V). The conductivity of the nanostructures increases when a negative voltage is applied to the gate electrode, showing that the nanofibres or nanoflakes are p-type semiconductors. It is seen from Figure 5a that a lower *V*_DS_ is needed to conduct a current in the nanostructures grown on the wide electrodes (nanofibers) compared to the structures grown on the narrow electrodes (nanoflakes), as was also seen in Figure 3b. We explain this difference in turn-on voltage by the fact that the nanofibers have a larger contact area to the gold electrode surface compared to the nanoflakes and therefore have a smaller contact resistance. We have not observed any saturation behavior even when the drain–source voltage is swept up to 70 V (at this voltage, the fibers and flakes are destroyed); however, this is as expected since the electrode gap width to oxide thickness ratio is too small. Therefore, the field-effect mobility and the threshold voltage cannot be extracted from the data. The curves sweeping back to 0 V (Figure 5) show hysteresis. This effect is often observed for organic semiconductors [27] and is most likely due to charge trapping.

The transfer (*I*_DS_ vs. *V*_DS_) curves for the devices containing nanoflakes are shown in Figure 5b. The sweeps were made by sweeping the gate voltage from 0 to 20 V, back to -20 V, returning to 0 V. The drain–source voltage was kept constant at 0 and -20 V, respectively. These curves confirm that the nanoflakes behave as p-type semiconductors and exhibit an onset voltage of around -10 V.

## Conclusions and Outlook

A method for growing in situ organic nanoflakes or nanofibers on a transistor platform was demonstrated in this work. The results show that the morphology of the nanostructures is strongly influenced by the drain and source electrodes width. When the gold electrodes are narrow, the *p*-6P molecules form high and narrow flake-like structures. This could be explained by topographical boundaries for *p*-6P diffusion on these substrates, which leads to more vertical aggregation. On wider gold electrodes, the larger surface diffusion areas lead to nanofiber growth, similar to growth on plane gold surfaces [21] (faceted morphology). The nanofibers start to conduct electrical current at lower *V*_DS_ than the nanoflakes. The proposed explanation for this behavior is that the *p*-6P nanofibers have a bigger contact area with the electrodes, which reduces the contact resistance. Both kinds of structures present typical electrical characteristics of an organic semiconductor connected to a metal electrode in which a current starts to flow after application of a certain voltage.

Investigations of the maximum bridgeable gap were performed and showed a probability of 1 for nanostructures bridging a gap of 200 nm. Since our oxide thickness is limited to 100 nm, the ratio between gap and insulator thickness is not high enough for the nanostructure transistor to reach saturation. However, electrical measurements show that an applied negative gate voltage increases the conductivity and thereby confirms that holes are the primary charge carriers.

The ability to pattern in situ growth of organic nanofibers and nanoflakes on device platforms opens up a route for use in a wide range of applications, such as large-scale fabrication of OLEDs and OFETs. We have demonstrated how the electrical properties of these structures can be altered by changing the structures morphology. In addition, nanofibers can be relatively straightforward assembled from other organic molecules with different optoelectronic properties [28]. For better transistor performance, one possibility would be to employ a bottom-contact/top-gate geometry that increases the contact area from which carriers inject.
